# Integrated analysis of single-cell and bulk RNA-sequencing identifies a signature based on T-cell marker genes to predict prognosis and therapeutic response in lung squamous cell carcinoma

**DOI:** 10.3389/fimmu.2022.992990

**Published:** 2022-10-14

**Authors:** Xuezhong Shi, Ani Dong, Xiaocan Jia, Guowei Zheng, Nana Wang, Yuping Wang, Chaojun Yang, Jie Lu, Yongli Yang

**Affiliations:** Department of Epidemiology and Biostatistics, College of Public Health, Zhengzhou University, Zhengzhou, China

**Keywords:** single-cell RNA-sequencing, T-cell marker genes, immunotherapy, lung squamous carcinoma, prognostic signature

## Abstract

Cancer immunotherapy is an increasingly successful strategy for treating patients with advanced or conventionally drug-resistant cancers. T cells have been proved to play important roles in anti-tumor and tumor microenvironment shaping, while these roles have not been explained in lung squamous cell carcinoma (LUSC). In this study, we first performed a comprehensive analysis of single-cell RNA sequencing (scRNA-seq) data from the gene expression omnibus (GEO) database to identify 72 T-cell marker genes. Subsequently, we constructed a 5-gene prognostic signature in the training cohort based on the T-cell marker genes from the cancer genome atlas (TCGA) database, which was further validated in the testing cohort and GEO cohort. The areas under the receiver operating characteristic curve at 1-, 3-, and 5-years were 0.614, 0.713 and 0.702 in the training cohort, 0.669, 0.603 and 0.645 in the testing cohort, 0.661, 0.628 and 0.590 in the GEO cohort, respectively. Furthermore, we created a highly reliable nomogram to facilitate clinical application. Gene set enrichment analysis showed that immune-related pathways were mainly enriched in the high-risk group. Tumor immune microenvironment indicated that high-risk group exhibited higher immune score, stromal score, and immune cell infiltration levels. Moreover, genes of the immune checkpoints and human leukocyte antigen family were all overexpressed in high-risk group. Drug sensitivity revealed that low-risk group was sensitive to 8 chemotherapeutic drugs and high-risk group to 4 chemotherapeutic drugs. In short, our study reveals a novel prognostic signature based on T-cell marker genes, which provides a new target and theoretical support for LUSC patients.

## Introduction

Lung cancer is one of the most common malignant tumors worldwide, with a high incidence and mortality rate (1). Lung squamous carcinoma (LUSC) is one of the major histological types of lung cancer, accounting for approximately 25% to 30% of all lung cancer cases (2). Although some drugs have been approved for the treatment of LUSC, the 5-year overall survival (OS) rate remains under 18% and most patients eventually develop drug resistance (3, 4). In recent years, immunotherapy has emerged as a promising strategy for cancer treatment, but only a minority of LUSC patients could benefit from immune checkpoint inhibitors (ICIs) treatment (5, 6). Therefore, it is very urgent to find suitable biomarkers to predict the prognosis and treatment response of LUSC.

The tumor microenvironment (TME) plays an important role in tumor progression and invasion (7). It has a critical impact for immunotherapy, which in turn can affect patient survival (8). In the TME, the development of novel cancer immunotherapies requires an in-depth understanding of tumor-resident T cells (9). T cells are known to play a major role in immunosurveillance and tumor eradication. The lack of T cells in tumors can lead to immunotherapy resistance (10). In addition, the success of chimeric antigen receptor T cell infusions in patients with leukemia and lymphoma also demonstrates the importance of T cells in antitumor immunity (11, 12). It has been reported that the presence of T cells plays an important role in the survival of non-small cell lung cancer (NSCLC). The presence and activation status of T cells can be used as a marker of the prognosis in NSCLC (13). As the anti-tumor immunity of T cells are poorly studied in LUSC, it is necessary to explore the gene expression profile of T cells and its relationship with prognosis and treatment response.

Single-cell RNA sequencing (scRNA-seq) has been of great significance for the development of targeted therapy and immunotherapy (14). In recent years, scRNA-seq reveals distinct immune cell subpopulations in TME, providing a new way to define functional biomarkers (15). Given this advantage, many studies have focused on identifying novel biomarkers for cancer by integrating scRNA-seq and bulk RNA-seq data (16–18). In our study, we performed an integrative analysis of scRNA-seq and bulk RNA-seq of LUSC to identify T-cell marker genes and construct a prognostic signature in the training cohort. The test and gene expression omnibus (GEO) cohort were used to further evaluate the predictive power of the signature. In addition, we analyzed the differences in tumor immune microenvironment (TIME), tumor mutational burden (TMB) and drug sensitivity between the two risk groups. We believe that our findings will provide potential prognostic biomarkers and therapeutic targets for LUSC.

## Materials and methods

### scRNA-seq data and transcriptome data acquisition

The scRNA-seq data of 2 purification LUSC tumor samples (GSM3635278 and GSM3635285) of GSE127465 were downloaded from the GEO database (http://www.ncbi.nlm.nih.gov/geo/). The bulk RNA-sequencing data and clinical information of LUSC patients were downloaded from the cancer genome atlas (TCGA) database (https://portal.gdc.cancer.gov), including 49 normal patients and 502 tumor patients. After excluding tumor patients without survival data, 488 tumor patients were included in the analysis. In addition, GSE37745 (n =66), GSE73403 (n =69) and GSE74777 (n =107) were downloaded from the GEO dataset to validate the prognostic power of the model. Data from GEO were integrated into the entire set and batch effects were corrected with the “ComBat” algorithm of “sva” package (19). The bulk RNA-sequencing data were processed with log2 transformation.

### scRNA-seq data analysis

The R software “Seurat package” (20) was used to convert scRNA-seq data into Seurat objects. Firstly, we performed quality control the scRNA-seq data by removing clusters with cell counts less than 3, cells with the number of genes mapped less than 50 and cells with more than 5% of mitochondrial genes. Then, the “NormalizeData” package was applied for data normalization. The top 15 principal components (PCs) were extracted by principal component analysis (PCA) based on the top 2,000 highly variable genes. T-distributed stochastic neighbor embedding (t-SNE) was used for unsupervised clustering and unbiased visualization of cell subpopulations on a two-dimensional map (21). The “FindAllMarkers” function was used to compare the differences of gene expression between a cluster and all other clusters. To identify the marker genes for each cluster, |log2 (fold change) | > 1 and adjusted *P*-value< 0.05 were used. Finally, the “SingleR” package (22) was used to annotate the cell subpopulations of the different clusters.

### Construction and validation of a prognostic signature

We integrated transcriptomic and survival data from 488 LUSC patients and randomly divided them into training and testing cohort in a 7:3 ratio. The univariate Cox regression analysis was performed to identify the T-cell marker genes that were significantly associated with prognosis in the training cohort (*P*<0.05). Subsequently, the least absolute shrinkage and selection operator (LASSO) Cox regression was performed to select the optimal λ to incorporate into the model. Finally, the selected key genes were included in multivariate Cox regression analysis. T-cell marker genes risk score (TCMGrisk) calculating formula was:


$$TCMGrisk=\sum_{i=1}^{n}co\text{e}f_{i}*x_{i}$$

where coef_i_ means the coefficients, *x_i_* is the FPKM value of each T-cell marker genes. The patients were divided into high-risk and low-risk groups based on the median value of the TCMGrisk. Kaplan-Meier (K-M) curve was used to evaluate the differences of overall survival (OS) between two groups. The time-dependent receiver operating characteristic (ROC) curves and the area under curve (AUC) were measured by package “survivalROC” in R software, which was used to evaluate the prognostic predictive accuracy of the model. We used the same method to validate the model predictive power on the testing cohort and GEO cohort.

### Exploration of mRNA and protein expression levels of signature genes

We studied 49 pairs of LUSC tumor patients and normal patients from the TCGA database to compare the differences in the mRNA expression levels of signature genes. The immunohistochemistry (IHC) staining images of the signature genes were obtained from the human protein atlas database (HPA; https://www.proteinatlas.org/), which was a valuable database that contains IHC-based expression data for the 20 most common cancer (23).

### Relationship between prognostic signature and clinicopathological factors

To facilitate clinical application and provide a more convenient tool for predicting the prognosis of LUSC patients, we integrated TCMGrisk and clinical factors including age, gender, stage to construct the nomogram. Furthermore, calibration curves were plotted to assess the agreement between actual and predicted values with the 45° dotted line indicating the optimal prediction. Decision curve analysis (DCA) was used to assess the net clinical benefit of TCMGrisk and clinical factors on patient survival outcomes (24).

### Gene set enrichment analysis

Gene set enrichment analysis (GSEA) (25) was used to assess related pathways and molecular mechanisms between low-risk and high-risk groups of LUSC patients. Kyoto encyclopedia of genes and genomes (KEGG) gene sets and phenotype tag expression files were loaded into the GSEA software and run 1,000 times to demonstrate function consistently. The screening criteria were |normalized enrichment score (NES)| > 1, nominal (NOM) *P*-value< 0.05 and FDR *q*-value< 0.25.

### Evaluation of tumor immune microenvironment

The immune score, stromal score and ESTIMATE score of LUSC patients were assessed with the estimation of stromal and immune cells in malignant tumor tissues using expression data (ESTIMATE) algorithm (26). The levels of 22 immune cell infiltration were assessed with the cell type identification by estimating relative subsets of RNA transcripts (CIBERSORT) algorithm (27). The activity of immune cell and immune function of each sample was calculated by single sample GSEA (ssGSEA). Marker genes for different immune cells were obtained from previous studies (28) and listed in **Table S1**. Wilcoxon test was used to estimate differences in the expression levels of immune checkpoints and human leukocyte antigen (HLA) -related genes between low-risk and high-risk groups. Finally, we retrieved the tumor immune dysfunction and exclusion (TIDE) score file from the TIDE website (29) (http://tide.dfci.harvard.edu). We then assessed the difference in immune checkpoint blockade response between the two groups using the “ggpubr” package.

### Mutation and drug sensitivity analysis

The somatic mutation data from LUSC patients were downloaded from the TCGA database. The number of mutation frequencies and exon lengths were calculated for each patient. To identify gene mutation characteristics between different risk groups, waterfall plots were generated using the “maftools” package and TMB value were described. We divided LUSC patients into low-TMB group and high-TMB group based on median TMB value. Wilcoxon test was used to compare the differences of the TMB value between the two groups. K-M curve was used to evaluate the differences of OS between the two groups. The chemotherapeutic response of LUSC patients was assessed by genomics of drug sensitivity in cancer (GDSC) (30). We utilized “pRRophetic” package (31) to assess the chemotherapeutic response based on the 50% maximum inhibitory concentration (IC50).

### Statistical analysis

All statistical analyses were conducted using the R software version 4.1.3 (http://www.R-project.org). Unless otherwise noted, *P*< 0.05 was considered as statistical significance.

## Results

### Identification of T-cell marker genes expression profiles

**Figure 1:**
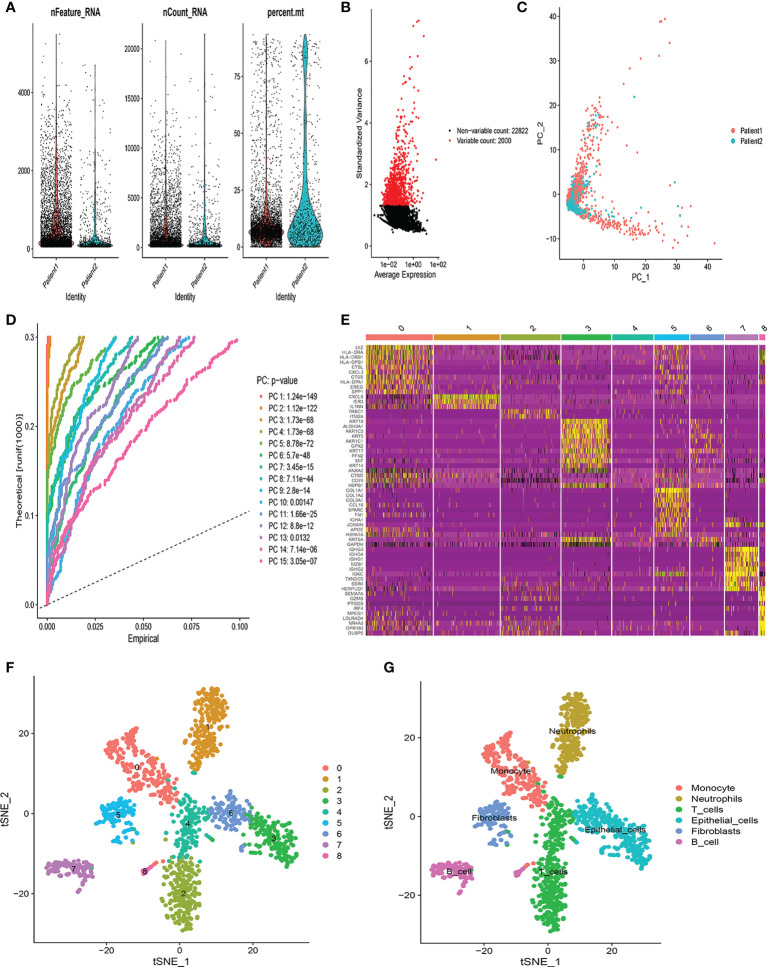
Identification of T-cell marker genes by scRNA-seq analysis. **(A)** Quality control of scRNA-seq data from two LUSC samples. **(B)** The variance plot showed 22,822 genes in all cells, red dots represent the top 2000 highly variable genes. **(C)** PCA was used for dimensionality reduction. **(D)** 15 PCs were identified based on *P*-value< 0.05. **(E)** The heatmap showed the relative expression of genes in 9 clusters. Yellow represents high expressed genes and purple represents low expressed genes. **(F)** 9 clusters were visualized based on the t-SNE algorithm. **(G)** Cell subpopulations identified by marker genes.

The flow chart of this study was shown in **Figure S1**. The scRNA-seq data used in this study were obtained from 12,950 cells of 2 LUSC tumor samples. **Figure 1A** showed the range of detected gene numbers, the depth of sequencing and the percentage of mitochondrial content in each sample. After strict quality control filtering to remove low-quality cells, 1,370 cells were included in the subsequent analysis. After normalizing the data, we selected the top 2,000 highly variable genes (**Figure 1B**). The PCA method was used for dimensionality reduction (**Figure 1C**), and 15 PCs with *P*-value< 0.05 were selected for further analysis (**Figure 1D**). We identified a total of 1,086 differentially expressed marker genes from 9 clusters and listed in **Table S2**. The relative expression of marker genes in each cluster were presented in the heatmap (**Figure 1E**). Afterwards, the 9 clusters were visualized using the t-SNE algorithm (**Figure 1F**). Using the “singleR” algorithm to annotate cell subpopulations, we found that clusters 2 and 4 were defined as T-cells subpopulations (**Figure 1G**). Ultimately, we obtained 72 T-cell marker genes of LUSC according to |logFC| >1 and adjusted *P*-value< 0.05.

### Construction and validation of prognostic model

**Figure 2:**
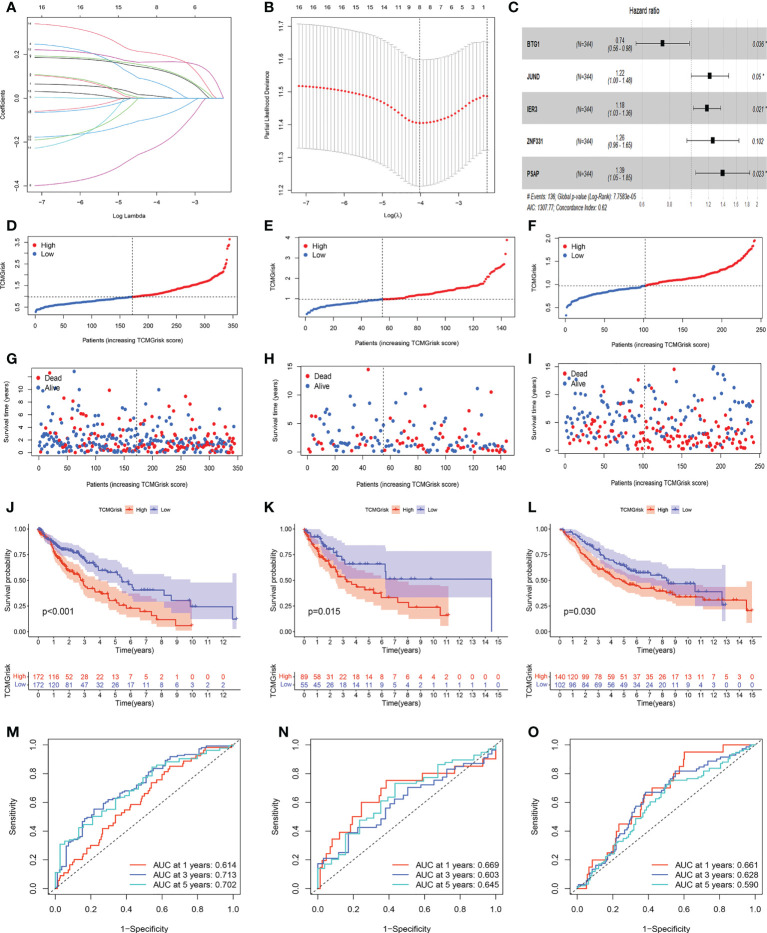
Construction and validation of prognostic models. **(A, B) **LASSO regression analysis. **(C)** Forest plot of multivariate Cox regression result, * represents P< 0.05. **(D–F)** Distribution of TCMGrisk score in training cohort, testing cohort and GEO cohort, respective. **(G–I)** Scatter plot of the OS of each patient in the training cohort, testing cohort and GEO cohort, respective. **(J–L)** The Kaplan-Meier curves in the training cohort, testing cohort and GEO cohort, respective. **(M–O)** The AUC at 1-, 3-, and 5-years of prognostic models in the training cohort, testing cohort and GEO cohort,respective.

Through univariate Cox regression analysis, we found that 17 T-cell marker genes were significantly associated with prognosis in the training cohort (*P*<0.05). (**Table S3**). LASSO analysis determined 8 genes based on the optimal lambda value and the corresponding coefficients (**Figures 2A, B**). Multivariate Cox regression analysis obtained 5 genes, including BTG1, JUND, IER3, ZNF331 and PSAP (**Figure 2C**). Based on their correlation coefficients, a TCMGrisk was built: TCMGrisk  =  (-0.297× BTG1expression) +  (0.197 × JUNDexpression) +  (0.166 × IER3expression) +  (0.228 ×ZNF331expression) +  (0.330×PSAPexpression). The patients were divided into high-risk and low-risk groups based on the median TCMGrisk (median TCMGrisk =0.973). The scatter plot of TCMGrisk indicated that as TCMGrisk score increased, OS decreased while mortality rise (**Figures 2D–I**). Subsequently, we assessed the prognostic value of the model. Compared with the low-risk group, the high -risk group had significantly longer survival (*P*<0.001) (**Figure 2J**). The AUC of 1-, 3- and 5-years of training cohort were 0.614, 0.713 and 0.702, respectively (**Figure 2M**). To further verify the robustness of the model, we perform the same analysis in the test cohort and GEO cohort. The results of the test cohort showed that OS of the low-risk group was better than that of the high-risk group (*P*=0.015) (**Figure 2K**). The AUC in 1-, 3- and 5-years were 0.669, 0.603 and 0.645, respectively (**Figure 2N**). The results of the GEO cohort showed that OS of the low-risk group was better than that of the high-risk group (*P*=0.030) (**Figure 2L**). The AUC in 1-, 3- and 5-years were 0.661, 0.628 and 0.590, respectively (**Figure 2O**). Both results showed that the model had a good predictive power.

### Differential expressions of signature genes

We examined the mRNA and protein expression levels of signature genes. IER3 expression was upregulated in LUSC patients, whereas JUND, PSAP and ZNF331 expression was downregulated in LUSC patients when compared with normal patients. BTG1 expression was not statistically significant between the two groups (**Figure S2A–S2E**). IHC results from the HPA database were used to further evaluate the expression of signature genes in LUSC. IER3 protein was significantly highly expressed in LUSC tissue, with strong antibody staining and more stained cells. While JUND and PSAP proteins were significantly highly expressed in normal tissue (**Figures S2F–H**). ZNF331 and BTG1 were not shown in HPA databases.

### The establishment of nomogram and decision curve analysis

**Figure 3:**
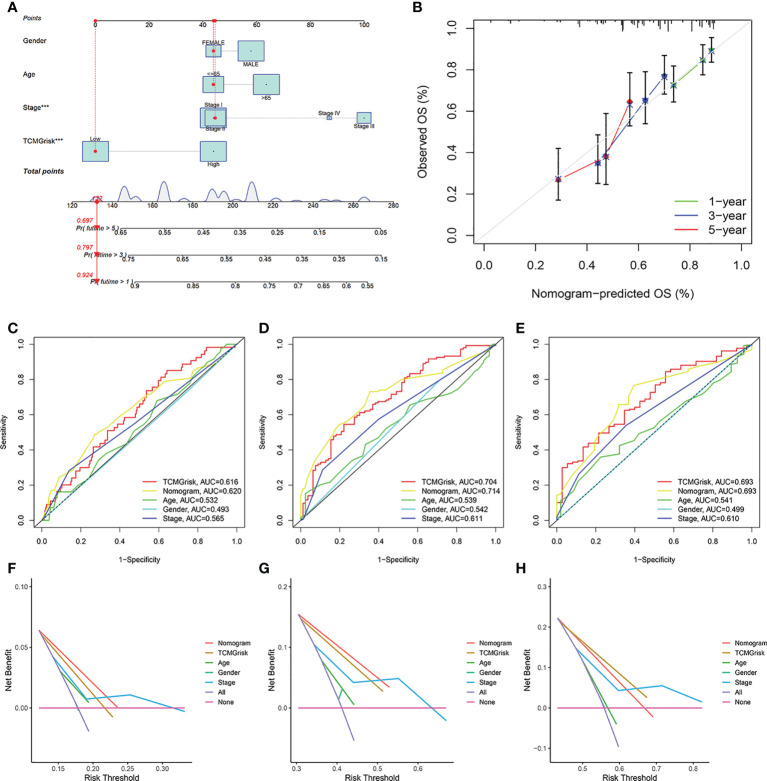
The establishment and validation of nomogram. **(A)** The construction of the nomogram. **(B)** Calibration curve for assess the agreement at 1-, 3- and 5-year OS. **(C–E)** The AUC of the nomograms compared for 1-, 3-, and 5-year OS, respective. **(F–H)** The DCA curves of the nomograms compared for 1-, 3-, and 5-year OS, respective.

We constructed a nomogram by integrating clinical factors and TCMGrisk to predict 1-, 3-, and 5-year survival probabilities of LUSC patients, respectively (**Figure 3A**). Calibration plots showed that the observed values were highly consistent with the predicted values (**Figure 3B**). In addition, The AUCs exhibited the nomograms held more clinical net benefit in predicting prognosis at 1-, 3-, and 5-years (**Figures 3C–E**). DCA showed that the nomogram provided the optimum clinical net benefit for 1- and 3-year OS but not for 5-year OS as well (**Figures 3F–H**). This suggested that the nomogram based on TCMGrisk could be used as an effective method to predict prognosis of patients in clinical practice.

### Gene set enrichment analysis

**Figure 4:**
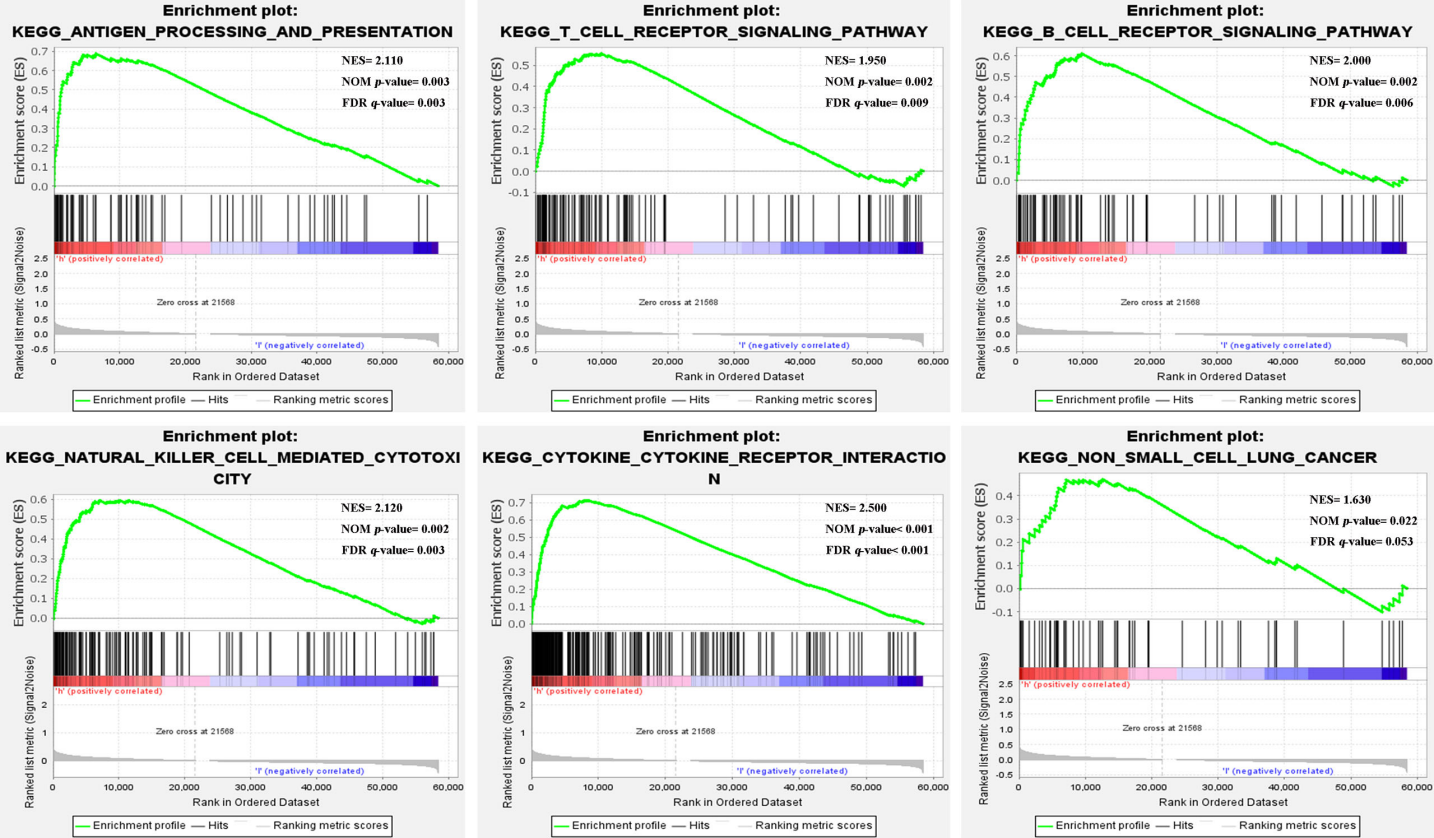
Gene set enrichment analysis.

The GSEA results showed that the high-risk group from the training cohort was mainly enriched in immune-related pathways (nom-*P*<0.05), such as antigen processing and presentation, T cell, B cell, Natural killer cell, and cytokine receptor interaction signaling pathway etc. Notably, the high-risk group was enriched in the non-small cell lung cancer pathway (**Figure 4**). Because these biological pathways were immune-related and involved in tumor immunity, we further analyzed immunity to compare the differences between the two groups.

### Estimation of tumor immune microenvironment and immune-related genes

**Figure 5:**
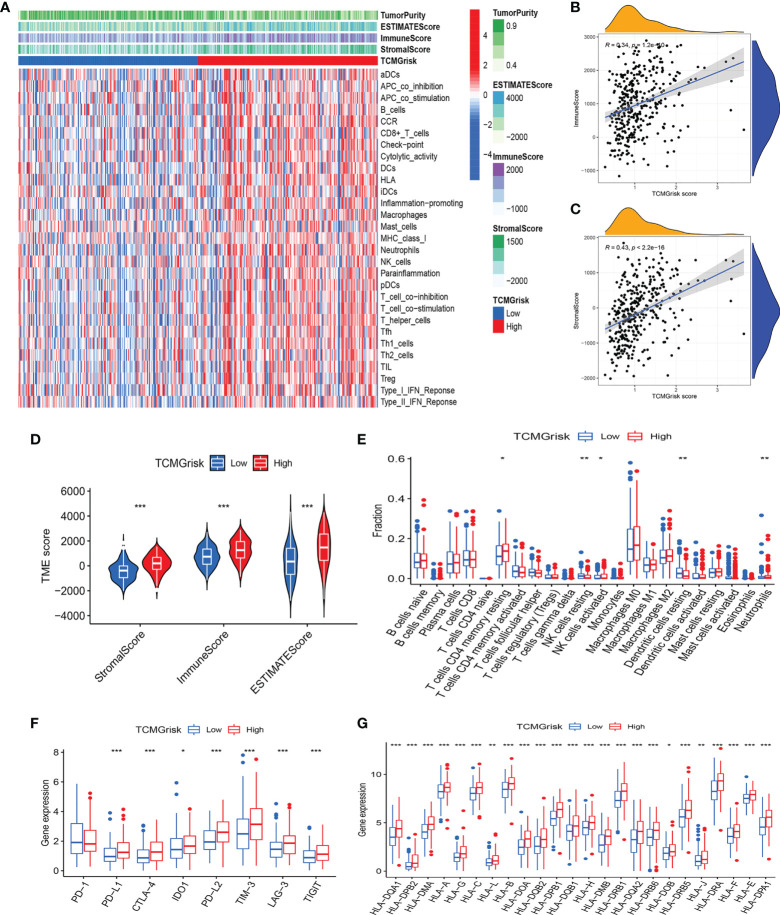
Characteristics of tumor immune microenvironment. **(A)** Heatmap showed the relationship between TCMGrisk and 22 immune-related functions. **(B)** Correlation between TCMGrisk and immune score. **(C)** Correlation between TCMGrisk and stromal score. **(D)** Differences expression levels of stromal, immune, and ESTIMATE scores between low-risk and high-risk groups. **(E)** Difference expression levels of 22 types of tumor-infiltrating immune cells between low-risk and high-risk groups. **(F)** Differential expression levels of the immune checkpoint-related genes between low-risk and high-risk groups. **(G)** Differential expression levels of the human leukocyte antigen-related genes between low-risk and high-risk groups. ***P< 0.001, **P< 0.01, *P< 0.05.

We further investigated the relationship between TCMGrisk and TIME. The heatmap results indicated that immune-related functions were more active in the high-risk group (**Figure 5A**). Correlation analysis showed that TCMGrisk was positively correlated with immune score and stromal score (**Figures 5B, C**). The ESTIMATE algorithm results showed that the stromal score, immune score and estimated score were all significantly higher in the high-risk group (*P*<0.001) (**Figure 5D**). Results of the ssGSEA algorithm found that T cell CD4 memory resting, NK cell activated, dendritic cell resting, and neutrophils were highly expressed in the high-risk group (**Figure 5E**). Considering the role of ICIs in immunotherapy, we compared the expression levels of eight common immune checkpoint-related genes in the two risk groups. The results showed that PD-L1, CTLA-4, IDO1, PD-L2, TIM-3, LAG-3 and TIGIT were highly expressed in the high-risk group, while PD-1 expression was not statistically different in the two risk groups (**Figure 5F**). Importantly, the TIDE score results showed that high-risk patients had significantly lower scores (**FigureS3**). In addition to this, we found that the expression levels of HLA-related genes were all higher in the high-risk group (**Figure 5G**). The above results suggested that the high-risk group may be more suitable for ICIs therapy.

### Gene mutation analysis

**Figure 6:**
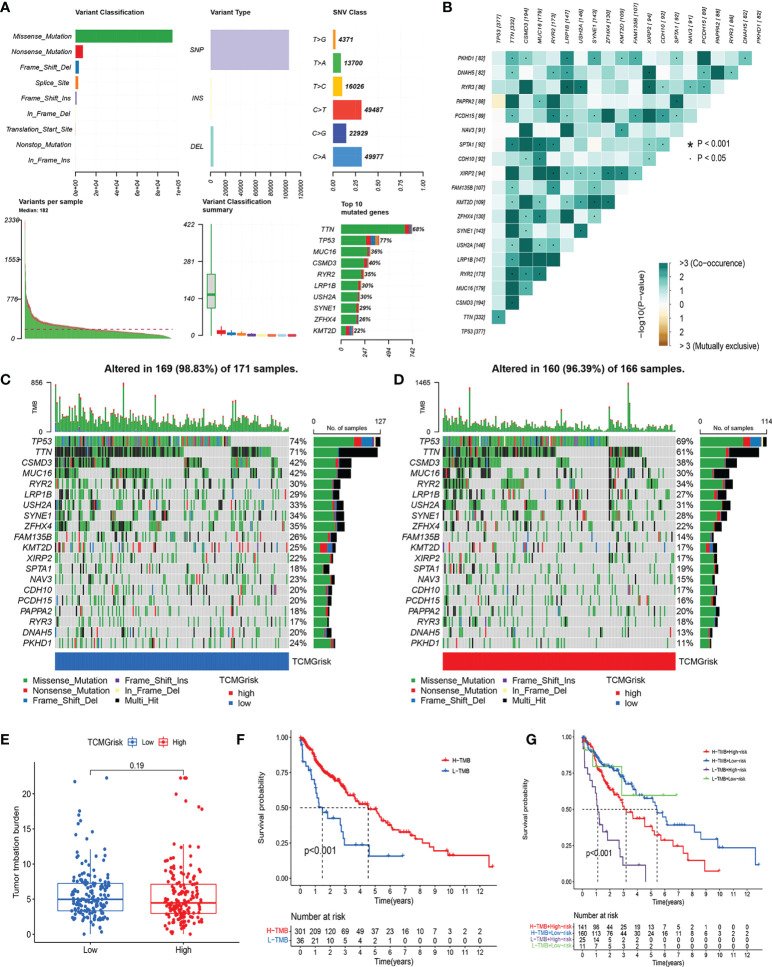
Characteristics of somatic mutations. **(A)** The overall mutation profile of LUSC. **(B)** Interaction effect of genes mutating differentially in patients in the low-risk and high-risk groups. **(C)** The mutation frequency of genes in the low-risk group. **(D)** The mutation frequency of genes in the high-risk group. **(E)** Differential expression levels of TMB between low-risk and high-risk groups. **(F)** The Kaplan-Meier curves for the low-TMB and high-TMB groups. **(G)** The Kaplan-Meier analysis curves for the patients stratified by TCMGrisk and TMB.

The overall mutation profile of LUSC was shown in **Figure 6A**. **Figure 6B** demonstrated the interaction of genetic mutations, with co-occurrence of mutations between most genes (*P*<0.05). In addition, we also investigated the genetic mutations in the low-risk and high-risk groups. We found that TP53, TTN, and CSMD3 were the most frequently mutated genes in the low-risk and high-risk groups (**Figures 6C, D**). There was no difference in TMB expression levels between the two risk groups (*P*=0.19) (**Figure 6E**). The K-M curves showed that high-TMB group had a better prognosis than low-TMB group (*P*<0.001) (**Figure 6F**). After combining our model, the prognosis of the low-risk+ high-TMB group was significantly better than that of the high-risk+ low-TMB group (*P*<0.001) (**Figure 6G**).

### Drug sensitivity analysis

**Figure 7:**
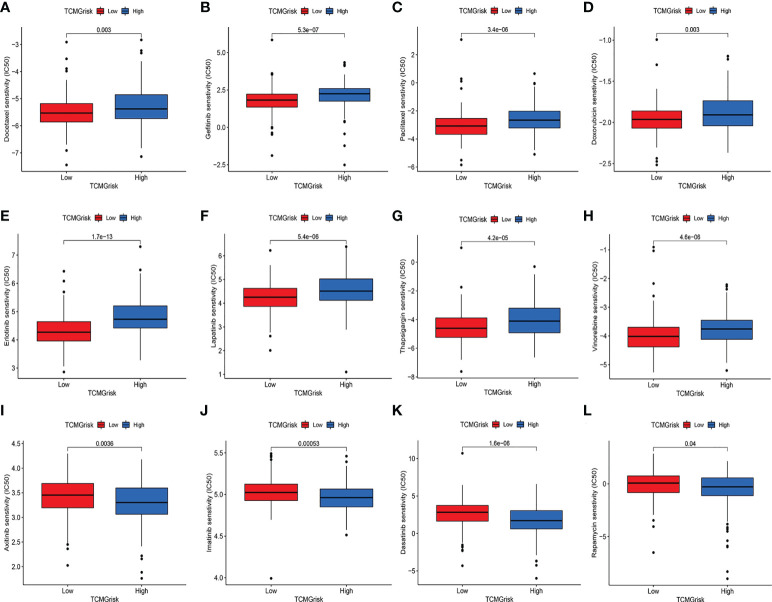
Evaluation of drug sensitivity. The comparisons in IC50 value of Docetaxel **(A)**, Gefitinib **(B)**, Paclitaxel **(C)**, Doxorubicin **(D)**, Erlotinib **(E)**, Lapatinib **(F)**, Thapsigargin **(G)**, Vinorelbine **(H)**, Axitinib **(I)**, Imatinib **(J)**, Dasatinib **(K)**, and Rapamycin **(L)** between low-risk and high-risk groups.

We further explored the difference in IC50 levels of chemotherapeutic drugs in the low-risk and high-risk groups (**Figures 7A–L**). The results found that patients in the low-risk group had lower IC50 for the anti-cancer drugs including docetaxel, gefitinib, paclitaxel, doxorubicin, erlotinib, lapatinib, thapsigargin, and vinorelbine. In contrast, patients in the high-risk group had lower IC50 for the anti-cancer drugs including axitinib, imatinib, dasatinib, and rapamycin. The above results suggested that TCMGrisk could be used as predictors for anti-cancer drug selection.

## Discussion

Immunotherapy has emerged as a powerful clinical strategy for the treatment of cancer. Recently, there has been renewed interest in the immunotherapy of lung cancer with the positive results of ICIs (32). However, exploring patients with LUSC who can benefit from immunotherapy remains a great challenge. The current study shows that scRNA-seq technology is a powerful tool for exploring tumor heterogeneity and different cell subpopulations, which is important for identifying potential therapeutic targets (33). In this study, we performed scRNA-seq analysis to explore T-cell marker genes in LUSC and construct a prognostic signature using the training cohort. The test and GEO cohort were used to further evaluate the predictive power of the signature. In addition, we found higher levels of immune score, stromal score, immune cell infiltration, immune checkpoints, and somatic mutations in the high-risk group. More immune-related pathways were also enriched in the high-risk group.

In this study, the prognostic signature was consisted of 5 T-cell marker genes, including BTG1, JUND, IER3, ZNF331 and PSAP. It was reported that BTG1 overexpression inhibited tumor cell proliferation, metastasis, invasion, and promoted apoptosis (34). The up-regulation of BTG1 in NSCLC reduced the migration and invasion of NSCLC cells by regulating the expression of CyclinD1, Bcl-2 and MMP-9 proteins, thus improving the prognosis of patients (35). JUND accelerated tumor growth, inhibited apoptosis and enhanced cancer cell invasion (36). It has been reported that the loss of JUND completely eliminates RAS-driven lung tumorigenesis (37). IER3 was an inhibitor of apoptosis, and higher expression of IER3 contributed to the progression of lung cancer (38). ZNF331 was a zinc finger protein whose overexpression is involved in transcriptional regulation. ZNF331 was found to be an oncogene and was associated with poor prognosis in many studies (39–41). The expression of PSAP in lung adenocarcinoma was higher than that in normal tissues, but further exploration the expression level and prognostic significance of PSAP was needed in LUSC (42). In addition, we explored the mRNA expression of signature genes, which was consistent with protein expression trends in LUSC and normal lung tissues. The signature genes identified in this study may provide potential molecular mechanisms for further clinical studies of LUSC.

The performance of the prognostic signature based on 5 T-cell marker genes was further validated in the testing cohort and the GEO cohort. We observed consistent results in both cohorts, indicating good robustness and reproducibility of the signature. Furthermore, we constructed a nomogram to visualize and predict patients’ 1 -, 3 -, and 5-year survival probabilities. Multiple validation methods (Calibration plots, AUC, and DCA) have shown that the nomogram had higher predictive accuracy. Therefore, this nomogram could guide the establishment of individualized examination procedures for LUSC patients and promote the effective use of medical resources.

Since TME plays a critical role in antitumor response and can significantly affect prognosis (43), we investigated the relationship between TCMGrisk and TME. First, we observed a significant increase in immune score, stromal score, and ESTIMATE score in the high-risk group compared to the low-risk group. Next, 22 immune cell infiltration levels also showed a higher proportion of CD4+ T cells resting, NK cells, dendritic cells and neutrophils in the high-risk group, suggesting that these patients may be in a relatively active state of anti-tumor immune response. In addition, ICIs as a potential therapeutic target for lung cancer (44). Our results showed that common immune checkpoint-related genes (PD-L1, CTLA-4, IDO1, PD-L2, TIM-3, LAG-3 and TIGIT) were highly expressed in the high-risk group, and TIDE was lowly expressed in high-risk group, suggesting that immunotherapy may be more suitable for high-risk group. Finally, HLA was a major histocompatibility complex (MHC) expression product in human, an antigen-presenting molecule that modulates the immune response in lung cancer (45, 46). The result showed that the all-HLA family genes were highly expressed in high-risk group, indicating that local immune response were more active. In conclusion, patients in the high-risk group exhibited more immune cell infiltration and immune response, suggesting that they were more likely to benefit from immunotherapy.

To better guide the treatment of LUSC, drug sensitivity analysis was performed in different risk groups. We investigated 12 anticancer drugs, including docetaxel, gefitinib, paclitaxel, doxorubicin, erlotinib, lapatinib, thapsigargin, vinorelbine, axitinib, imatinib, dasatinib, and rapamycin between low-risk and high-risk groups. The results showed that the low-risk group was sensitive to 8 anticancer drugs and the high-risk group was sensitive to 4 anticancer drugs, which provided a reference for clinical selection of chemotherapy drugs. In the follow-up study, we will further explore the clinical significance of these drugs with LUSC patients.

Although this study provided new insights to promote the development of new therapies for LUSC, it still had some limitations. First, all cohort studies were retrospective and require further validation in prospective cohort studies. Second, drug sensitivity needs further confirmation by cell experiment. Third, the number of scRNA-seq samples and the amount of data published in the public database was limited, thus the clinical and pathological parameters analyzed were not comprehensive, which may lead to potential biases. Therefore, it is necessary to carry out multi-center, large-sample, prospective double-blind trials for further verification in the future.

In conclusion, we developed a novel prognostic signature consisting of 5 T-cell marker genes by combining scRNA-seq and bulk RNA-seq data. Furthermore, the TCMGrisk was significantly associated with TIME, immune-related pathways and drug sensitivity. Our study provides new theoretical insights into the role of T-cell marker genes in the prognosis and precision therapy of LUSC patients.

## Data availability statement

The datasets presented in this study can be found in online repositories. The names of the repository/repositories and accession number(s) can be found in the article/**Supplementary Material**.

## Author contributions

XZS and AND designed this study. XZS, AND and GWZ were responsible for data collection. XZS and AND wrote this manuscript. XCJ, GWZ, NNW, YPW, CJY, JL and YLY reviewed and polished this research.

## Conflict of interest

The authors declare that the research was conducted in the absence of any commercial or financial relationships that could be construed as a potential conflict of interest.

## Publisher’s note

All claims expressed in this article are solely those of the authors and do not necessarily represent those of their affiliated organizations, or those of the publisher, the editors and the reviewers. Any product that may be evaluated in this article, or claim that may be made by its manufacturer, is not guaranteed or endorsed by the publisher.
