# Supplementary material for: Integrated analysis of single-cell and bulk RNA-sequencing identifies a signature based on T-cell marker genes to predict prognosis and therapeutic response in lung squamous cell carcinoma
Source: Front Immunol. 2022 Oct 14;13:992990. doi: 10.3389/fimmu.2022.992990 (PMC9614104; doi:10.3389/fimmu.2022.992990)
Supplement: Supplementary file 1 [file DataSheet_1.zip › Supplement file/Supplement figure.docx]

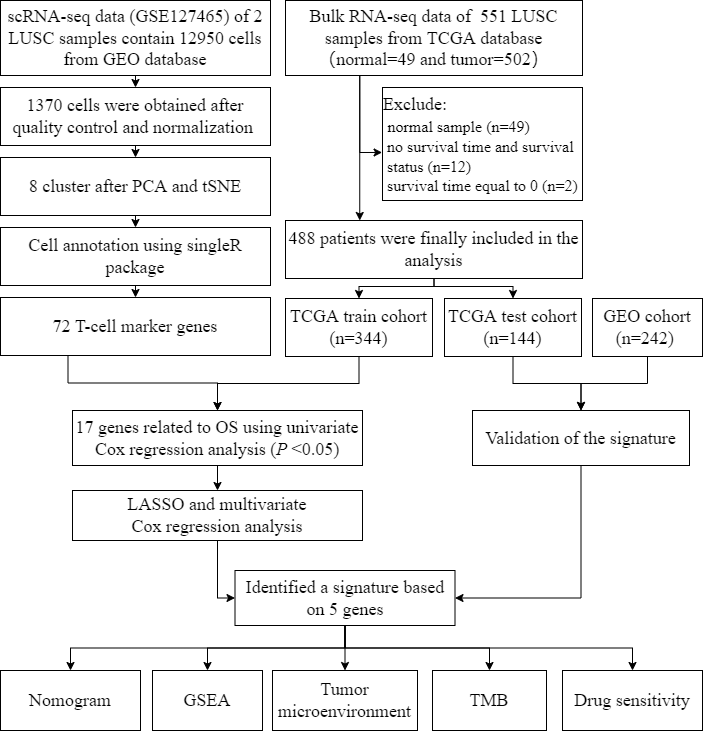


**Figure S1:** The flow chart of the study.

**Figure S2:** mRNA and protein expression levels of signature genes. **(A)** mRNA expression levels of signature genes in TCGA database. **(B)** Protein expression levels of signature genes in the HPA database.


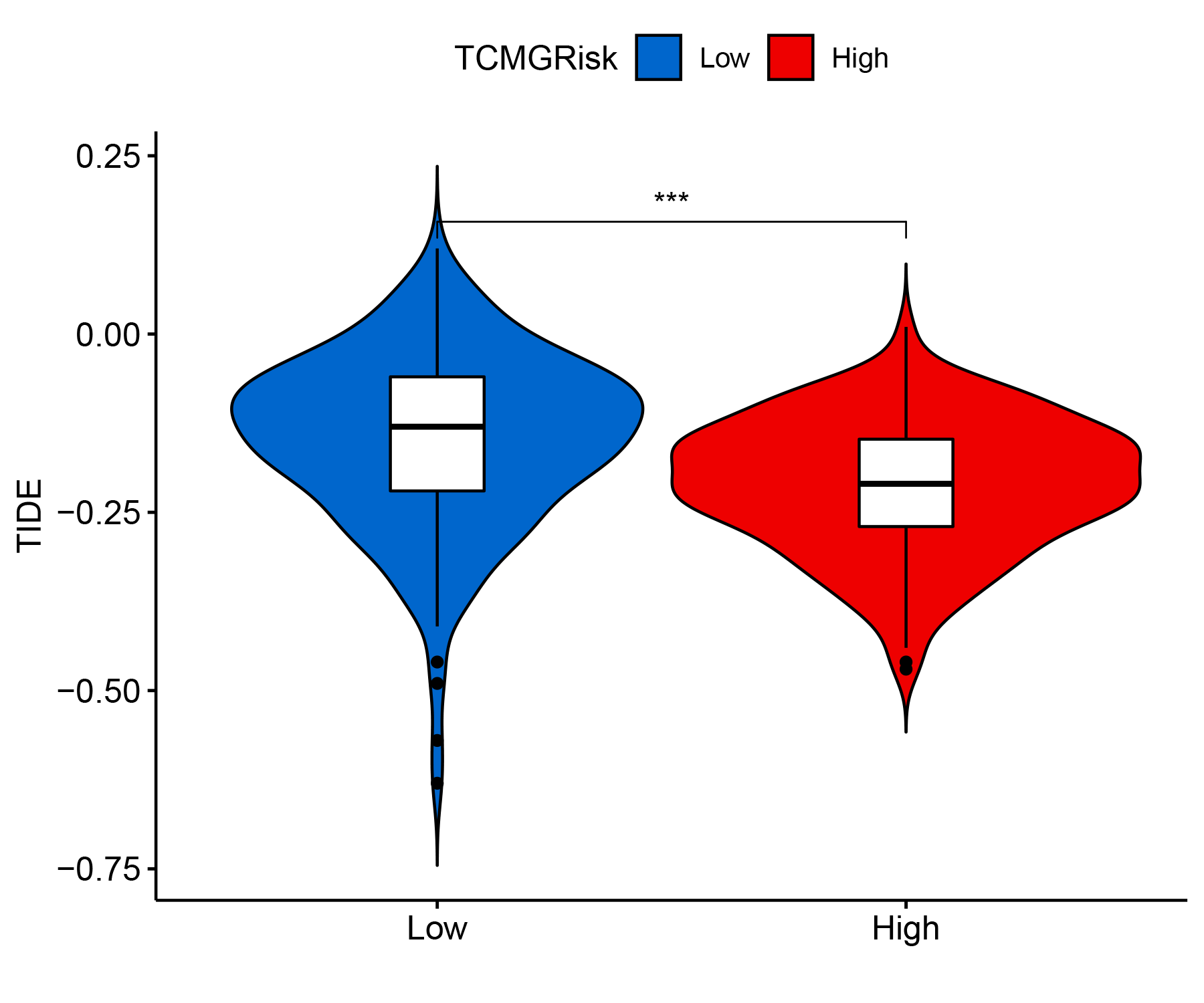


**Figure S3:** Comparisons of the tumor immune dysfunction and exclusion scores between low-risk and high-risk groups.
